# Genetically Engineered Mouse Models Support a Major Role of Immune Checkpoint-Dependent Immunosurveillance Escape in B-Cell Lymphomas 

**DOI:** 10.3389/fimmu.2021.669964

**Published:** 2021-05-25

**Authors:** Quentin Lemasson, Hussein Akil, Jean Feuillard, Christelle Vincent-Fabert

**Affiliations:** ^1^ UMR CNRS 7276/INSERM U1262 CRIBL, University of Limoges, Limoges, France; ^2^ Hematology Laboratory of Dupuytren Hospital University Center (CHU) of Limoges, Limoges, France

**Keywords:** B-cell lymphoma, immune surveillance, PD-1/PD-L1, CTLA-4, MHC-II, NKG2D

## Abstract

These last 20 years, research on immune tumor microenvironment led to identify some critical recurrent mechanisms used in cancer to escape immune response. Through immune checkpoints, which are cell surface molecules involved in the immune system control, it is now established that tumor cells are able to shutdown the immune response. Due to the complexity and heterogeneity of Non Hodgkin B-cell Lymphomas (NHBLs), it is difficult to understand the precise mechanisms of immune escape and to explain the mitigated effect of immune checkpoints blockade for their treatment. Because genetically engineered mouse models are very reliable tools to improve our understanding of molecular mechanisms involved in B-cell transformation and, at the same time, can be useful preclinical models to predict immune response, we reviewed hereafter some of these models that highlight the immune escape mechanisms of NHBLs and open perspectives on future therapies.

## Introduction

Non Hodgkin B-cell Lymphomas (NHBLs) are malignant neoplasms characterized by an abnormal expansion of clonal B lymphocytes. Despite being subdivided in more than 50 entities (1), most of them can be ascribed as indolent (low grade) or aggressive forms (high grade). Immune escape, also called immunomodulation or immunoediting, is a way by which tumors neutralize and/or subvert the host’s immune system to their advantage (2). The concept of host immune response against neoantigens has been demonstrated long time ago (3) and Tumor Infiltrated Lymphocytes (TILs) have been recognized very early as a biomarker of the immune response against cancer (4). However, tumor cells are able to evade immune response by developing an immunosuppressive microenvironment through the regulation of immune checkpoint protein expression, such proteins being essential in the negative control of activated immune cells. Various immunotherapy treatments aim to reactivate antitumor immune response by targeting specific immune-checkpoint proteins (5). Programmed cell-Death 1 (PD-1) and its ligand Programmed cell-Death Ligand 1 (PD-L1) are the most studied immune checkpoint proteins. If immune restoring therapies have given spectacular results in some solid cancers such as those of the lung, colon or melanoma, effects on B-cell lymphomas are mitigated (6). There is an urgent need to understand how tumor B cells escape immune surveillance. Thanks to genetic engineering, mouse models are globally useful for phenocopying human B-cell lymphomas and investigating the emergence and progression of transformed B cells as well as their concomitant immune escape and for researching new therapeutic strategies.

**Figure 1:**
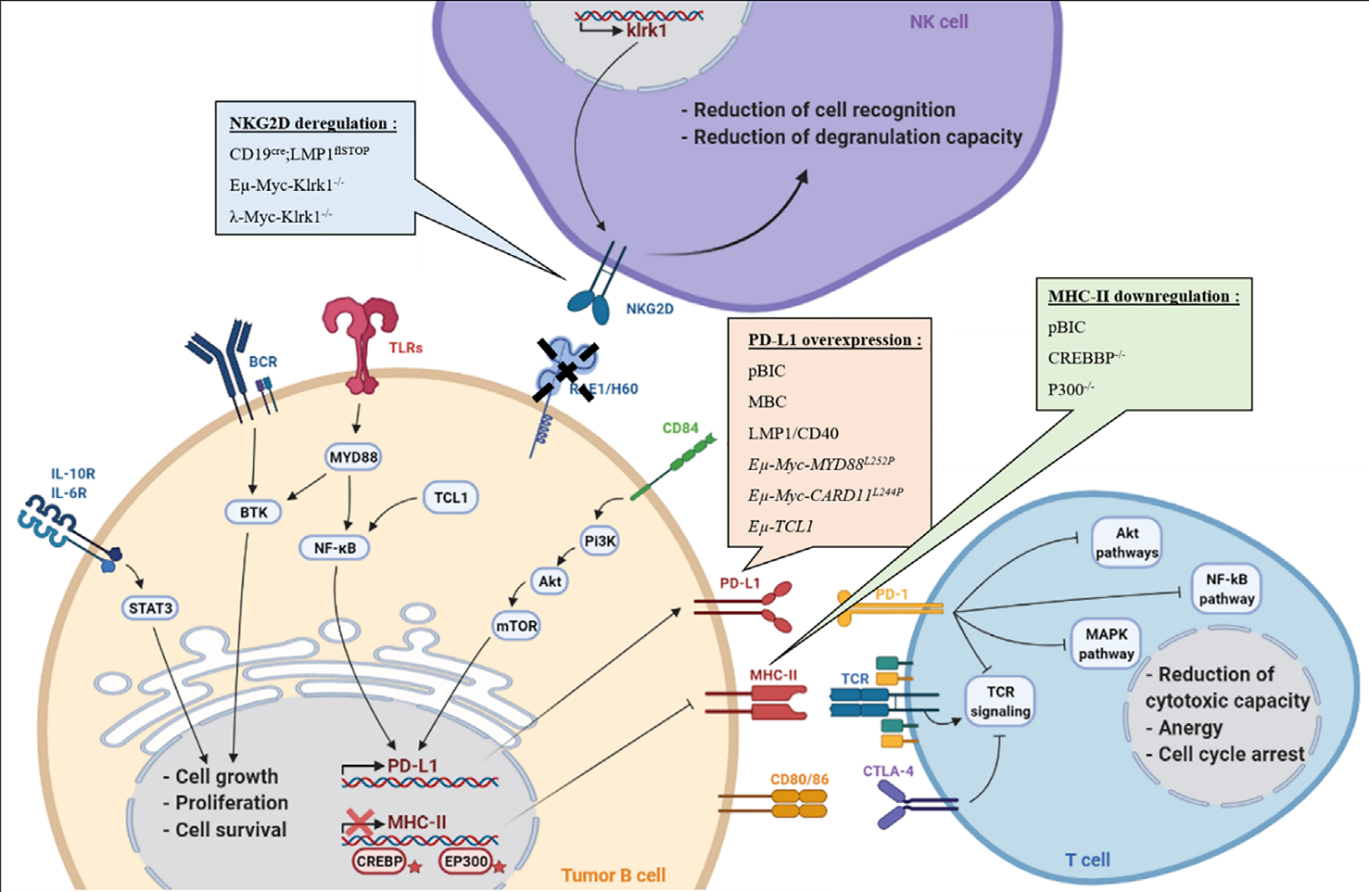
B/T/NK cells in the immune escape context. Representation of different mechanisms exploited by tumor B-cell lymphomas to escape immune control during lymphomagenesis in each presented transgenic mouse models: (i) overexpression of PD-L1 associated with MHC-II downregulation to induce T-cell anergy, (ii) Reduction of RAE1/H60 expression to induce NK cells tolerance, (iii) CTLA-4 expressed by T-cells is targeted by lymphoma B cells, leading to T-cell inactivation.

Here, we discuss different mouse models that have been used to study the B-cell immune surveillance (**Figure 1** and **Table 1**). The human relevance of these models is first presented, and then, the experimental results are summarized and discussed. A large part of studies presented hereafter investigated the PD-1/PD-L1 axis. However, other molecules such as CTLA-4, MHC-II or NKG2D are discussed. Strikingly, these models provide functional clues to genetic abnormalities as well as immune evasion occurring in B-cell lymphomas, and recurrently highlight the role of NF-κB. Indeed, as reviewed recently, NF-κB plays a major role in immune checkpoint controls and directly regulates PD-L1 expression (17).

**Table 1:** Transgenic mouse models.

Model		Strategy	Expression/Induction	Phenocopy	Immune escape strategy	Reference
**CRE regulated expression**						
pBIC	NF-κB constitutive signaling *Blimp1* inactivation *p53* inactivation		GC-B cell compartment (Cγl^CRE^)	ABC-DLBCL	PD-L1 overexpression MHC-II downregulation	(7)
MBC	endogenous MYD88^L252P^ expression BCL2 overexpression		B-cell compartment (Cd19^CRE^)	ABC-DLBCL	PD-L1 overexpression	(8)
CREBBP^-/-^	CREBBP inactivation		GC-B cell compartment (AID^CRE^)	DLBCL	MHC-II deregulation	(9)
P300^-/-^	p300 inactivation		GC-B cell compartment (AID^CRE^)	DLBCL	MHC-II deregulation	(9)
CD19^cre^;LMP1^flSTOP^	EBV's LMP1 expression		B-cell compartment (Cd19^CRE^)	EBV driven lymphomas	NKG2D blocking	(10)
LMP1/CD40	CD40 constitutive signaling		B-cell compartment (Cd19^CRE^)	SMZL	PD-L1 overexpression	(11)
**EμMyc/λMyc regulated expression**						
Eμ-Myc-MYD88^L252P^	transplantation of Eμ-*Myc*-MYD88^L252P^		B cell lineage	ABC-DLBCL	PD-L1/PD-L2 overexpression	(12)
Eμ-Myc-CARD11^L244P^	transplantation of Eμ-*Myc*-CARD11^L244P^ HSCs		B cell lineage	ABC-DLBCL	PD-L1/PD-L2 overexpression	(12)
hCTLA4-FcγR^-/-^	Human CTLA4 expression FcγR inactivation		B cell lineage	CLL	CTLA4 deregulation	(13)
Eμ-Myc-Klrk1^-/-^	NKG2D inactivation		B cell lineage	MYC driven lymphomas	Absence of NKG2D	(14)
λ-Myc-Klrk1-/-	NKG2D inactivation		B cell lineage	BL	Absence of NKG2D	(15)
Eμ-TCL1	TCL1 overexpression		B cell lineage	CLL	PD-L1/PD-L2 overexpression	(16)

(ABC-)DLBCL, (Activated B-Cell) Diffuse Large B-Cell Lymphoma; EBV, Epstein Barr Virus; SMZL, Splenic Marginal Zone Lymphoma; CLL, Chronic Lymphocytic Leukemia; BL, Burkitt Lymphoma.

## Deregulation of PD-1/PD-L1 Axis in Mouse Models of B-Cell Lymphomas

Discovered in the nineties, PD-1 and PD-L1 remain the most promising immune checkpoint targets for cancer immunotherapy (18, 19). PD-L1 (B7-H1 or CD274) binds to its receptor PD-1 (CD279) on T cells (20). PD-L1 first promotes naïve T-cell expansion through IL-10 stimulation (19). Then, PD-1/PD-L1 signaling induces effective inhibition/exhaustion of T cells in the context of chronic antigen stimulation (20). Among effects, PD-1/PD-L1 interaction leads to a drastic inhibition of the T-cell receptor (TCR) signaling as well as AKT, ERK and NF-κB signaling pathways. Essential for the immune response control, this mechanism is adopted by tumor cells to escape immune surveillance. PD-L1 expression by tumor B cells has been found in various lymphoma subtypes such as Diffuse Large B-Cell Lymphomas (DLBCL), associated or not with the Epstein Barr virus (EBV). This tumor phenotype is usually associated with an intratumoral infiltration of PD1^+^ T-lymphocytes (PD1^+^ TILs) (21, 22), thus exhibiting an inflamed phenotype according to classification of Chen (23). The selection pressure exerted by the immune surveillance is probably one of the drivers of the emergence of B-cell clones since chromosomal alterations in the 9p24.1 region which harbors the *PDL1* and *PDL2* loci are found in most classical Hodgkin lymphomas (HL) as well as in 27% of non Germinal Center B (GCB) DLBCLs, both B-cell cancers strongly associated with NF-κB activation (24). Anti-PD-1/PD-L1 therapy has given promising results in HL (25). PD-L1 expression is found in 26-75% (26) of patients with higher expression in Activated B-Cell (ABC) DLBCL, presumably due to the constitutively activated NF-κB as it is in Epstein Barr Virus (EBV)-positive DLBCL (27). However, correlation between PD-L1 expression and the response to PD-1/PD-L1 therapy in DLBCLs remains controversial (28).

Recently, two studies dealing with NF-κB associated DLBCLs showed similar results. The first one used the MBC model, developed by Knittel *et al*., which develop aggressive DLBCL-like tumors (29). These mice harbor the MYD88^L252P^ mutation and overexpress the anti-apoptotic factor BCL2, two features found in 29% and 40% of ABC-DLBCL cases respectively (30, 31). Flümann et al. found a striking resemblance of MBC tumors with human ABC-DLBCLs, with a similar gene expression profile (8). They also demonstrated overexpression of *Pdl1* on MBC lymphoma B cells associated with exhausted infiltrating CD4^+^ and CD8^+^ T cells, highlighting the immune escape strategy used by tumor cells.

In the second study, Reimann *et al*. engineered an original ABC-DLBCL model based on the demonstrated cooperation between c-MYC and NF-κB (32). Hematopoietic Stem Cells (HSC) from Eµ-*Myc* mice were stably transduced with naturally occurring NF-κB mutants, among them MYD88^L252P^ or CARD11^L244P^. Transduced Eµ-*Myc* cells were injected into lethally irradiated strain-matched C57BL/6 recipients. All these allograft models supported MYC-driven B-cell lymphomagenesis through increased protection against apoptosis, but only MYD88^L252P^ or CARD11^L244P^ Eµ-*Myc* tumors resembled to human ABC-DLBCL, including expression of PD-L1 which was responsible for an exhausted T-cell phenotype (12).

In both studies, the authors treated their mouse model with anti-PD-1 antibodies, leading to a significant increase in overall mouse survival and inducing phenotypic changes in infiltrating CD4^+^ and CD8^+^ T cells with a restored proliferation potential. The combination of anti-PD-1 antibody and BCL2 inhibitor showed an additive effect in the MBC model. The anti-PD-1 treatment seems to be really effective regarding these models. But recent human clinical trial showed a completely different reality (6), raising the question of resistance to these therapies. Some clues may have been given by the pBIC model. This model associates *Tp53* deletion, enhanced NF-κB signaling due to an activated IKK2 mutant, and *Blimp1* inactivation that blocks plasma-cell differentiation. Pascual *et al*. showed that, in these mice, DLBCL-like tumors exhibited an ABC-DLBCL phenotype with FOXP1 dysregulation and overexpression of PD-L1 by tumor cells. This phenotype was associated with intra-tumor infiltration of PD-1^+^ CD8^+^ T cells. Exhibiting an exhausted phenotype, these TILs also expressed other inhibitory molecules such as LAG-3 and 2B4 (7). In this model, anti-PD-1 treatment did not show any effect on mouse survival and anti-CD20 treatment alone had limited impact. But combining both antibodies markedly improved mouse survival and resulted in tumor regression with clearance of PD-1 TILs, strongly suggesting that therapies targeting the PD1/PD-L1 axis could be used in combination with other well established treatments in DLBCLs.

Taken together, these different mouse models for ABC-DLBCLs suggest that PD-1/PD-L1 immune checkpoint upregulation is one of the main mechanisms of immunosurveillance escape in ABC-DLBCL. It also indicates that, even when poorly effective alone, anti PD-1/PD-L1 therapies may very well be able to improve the effect of other molecules that directly target tumor B cells.

To our knowledge, only four mouse models of indolent B-cell lymphoma have been reported. One is related to Chronic Lymphocytic Leukemia (CLL) with *TCL1* (originally described in the former CD4 T-CLL, now called T-cell prolymphocytic leukemia) as a driver oncogene (33) and three others to marginal zone lymphomas, involving *Traf3* deletion (34), *BCL10* deregulation (35) and CD40 signaling (11). The question of immune surveillance was addressed only in the TCL1 and CD40 mouse models. First, Hofbauher *et al*. demonstrated that development of a CLL-like disease was associated with a shift from naïve to memory phenotypes of both CD4^+^ and CD8^+^ T cells in Eµ-*TCL1* transgenic mice (16). In the context of aging, McClanahan *et al*. described in this model a PD-1 overexpression on CD8^+^ T cells with a defect in immune synapse formation, associated with specific PD-L1 overexpression on B cells (36). In the same model, Lewinsky *et al*. demonstrated the role of CD84, a member of the Signaling Lymphocyte Activating Molecule (SLAM) family known to bridge between CLL cells and their microenvironment (37). CD84 (SLAMF5) activation upregulates PD-L1 expression on CLL cells and stroma cells, through the AKT/mTOR pathway, and promotes PD-1 expression on T cells resulting in their exhaustion (38). In an adoptive transfer of Eµ-*TCL1* CLLs, PD-L1 blockade restored the antitumor immune response, in conjunction with an enhanced pro-inflammatory microenvironment (39). However, the question of the tumor microenvironment arises with this strategy. Adoptive transfer of Eµ-*TCL1* leukemic cells proves their ability to invade and proliferate in a healthy microenvironment by their own, but this development is independent of the tumor microenvironment, and consequently a real immune escape mechanism remains hard to identify here.

Being also associated with NF-κB activating mutations, splenic marginal zone lymphomas exhibit an inflamed phenotype (40, 41). In the model of Hömig-Hözel *et al*., B-cell specific continuous CD40 signaling is due to specific B-cell expression of a chimeric LMP1/CD40 protein composed of the membrane moiety of the LMP1 protein of EBV and the intracytoplasmic transducing moiety of CD40 (11). LMP1/CD40 mice develop indolent lymphomas with clonal expansion of spleen marginal zone B cells. The indolent phenotype was shown to be associated with an overexpression of PD-L1 on B cells, such expression depending on NF-κB, STAT3 and BCR pathways (42). In this model, T-cell depletion resulted in progression toward an aggressive tumor, suggesting that some immune surveillance was still exerted on indolent B-cell lymphoma (43). Indeed, crossing the LMP1/CD40 and λ -*Myc* mice (in which *Myc* is under the control of the Ig lambda locus) led to the development of aggressive B-cell tumors with an immunoblast phenotype and further PD-L1 expression (32). This study suggests that the PD-1/PD-L1 axis is requested for emergence of an indolent lymphoma as well as for its progression towards an aggressive form. Concomitantly, our group demonstrated a beneficial effect of anti-PD-L1 therapy in the LMP1/CD40 model, resulting in tumor regression and T-cell reactivation. Interestingly, we also demonstrated that therapies targeting other pathways, such as NF-κB, JAK/STAT or BCR signaling, were also able to reduce PD-L1 surface expression of tumor B cells (42). Like in mouse DLBCL models, these results suggest that, as long as they are expressed, PD-1 and/or PD-L1 blockade in indolent B-cell lymphomas may synergize with other therapeutic molecules, for example, the specific inhibitors of JAK/STAT pathway, ruxolitinib, or, of BTK, ibrutinib.

In essence, these different transgenic mouse models highlight the major role of PD-1/PD-L1 axis deregulation in aggressive lymphomas development, but also showed the involvement of this immune checkpoint in the transformation of indolent lymphomas as long as they express PD-L1. They also indicate that the level of PD-L1 expression may be a critical parameter for immune evasion and tumor progression and point on the interest of combined therapies that include anti-tumor immune restoration.

## Deregulation of Other Immune Checkpoints in Mouse Models of B-Cell Lymphomas

### CTLA-4

In Humans, a single nucleotide polymorphism located in the 3q13.33 region that harbors the CD80 and CD86 loci is associated with the risk of DLBCL, providing evidence for the role of immune function in the etiology of these lymphomas (44). The activating effects of CD80 and CD86 on CD28 are neutralized by the Cytotoxic T-Lymphocyte Antigen 4 (CTLA-4). CTLA-4, normally expressed on the surface of activated and regulatory T cells as well as B cells, even if weakly, is the second major immune checkpoint molecule. As CTLA-4 has a higher affinity to CD28 than for CD80 or CD86, it downregulates primary T-cell responses by interaction with B7 family members expressed on Antigen Presenting Cells (APCs) (45). CTLA-4 was shown to be abnormally expressed on B-cell lymphomas, and is notably a part of the CLL signature (46).

In the Eµ-*TCL1* mouse model, CTLA-4, which is expressed on CLL-like B cells, seems to promote STAT3 activation pathway through its dimerization with surface CD86 followed by its internalization, and thus acting as a costimulatory signal (47). By using the adoptive transfer strategy, Do *et al*. showed that specific CTLA4 blockade on tumor cells could affect leukemic progression (13).

### MHC-II

Besides their direct role in antibody secretion, B cells express MHC-II and are also APCs. B-cells are able to improve T-cell activation and proliferation through a process called B/T-cell cooperation (48, 49). In B cell lymphomas, loss of MHC-II contributes to the immune evasion resulting in a decrease of T-cell activity. This loss of surface MHC-II may be due to genetic alterations of the *MHC-II* region, including homozygous deletions (50). Malignant B cells can also downregulate the expression of *MHC-II* by various mechanisms (7, 9, 51) such as the downregulation of the MHC-II transcriptional activators class II transactivator (*CIITA*). The consequence of *MHC-II* down-regulation in tumor B-cell escape has been exemplified in two KO models for the Histone Acetyl-Transferases (HAT) *Crebbp* or *Ep300* (which deletions are found in 50% of human DLBCLs). In these models, development of aggressive B-cell tumors was related to the fact that CD4^+^ T cells were unable to interact with B cells because the latter had lost the expression of MHC-II which is normally up-regulated by these HAT (9). In addition, loss of MHC-II was also demonstrated in the pBIC mouse lymphoma model, suggesting a cooperation with the PD-1/PD-L1 in immunosurveillance escape (7). If there is no current treatment allowing a direct restoration of MHC-II at the surface of tumor cells, McClanahan *et al*. showed a restoration of MHC-II surface expression after anti-PD-L1 treatment in the CLL model Eµ-*TCL1* (39).

Thereby, studies on pBIC and Eµ-*TCL1* models converge to show a striking correlation between PD-L1 and MHC-II deregulation in different types of lymphomas. Nevertheless, these findings deserve to be more closely investigated to improve the comprehension of immune checkpoints deregulation in lymphomas.

### NKG2D

In Humans, cells infected by the EBV are under powerful immune surveillance by T and NK cells. Compromising this immune surveillance such as in AIDS or after organ transplantation, results in aggressive EBV-related B-cell lymphoproliferations. NK-cell functions could strongly be altered in DLBCLs that can be associated with resistance to rituximab-based therapies (52). Mouse Natural Killer Group 2 member D (NKG2D) is a receptor expressed by all cytotoxic lymphocytes, notably NK cells, activated CD8^+^ T cells and activated macrophages (53). Two murine NKG2D ligands have been described (RAE-1 and H60) (53–56). Interaction between NKG2D and its ligands induces PI3K-AKT signaling, leading to an increase in target recognition, cytotoxic activity and a reduction in sensitivity to cell apoptosis (57). NKG2D ligands are normally expressed at the surface of any virally infected/tumorigenic cells. Both mouse and human studies suggest that tumor cells evade NKG2D recognition either by downregulating NKG2D ligands expression (14, 58) or by secreting massively circulating NKG2D ligands resulting in a downregulation of NKG2D expression on cytotoxic cells (59–61). The occurrence of B-cell lymphomas is accelerated in the absence of NKG2D. In mice, Guerra *et al*. showed, in 2008, that the deletion of *Klrk1*, the gene encoding NKG2D, in Eµ-*Myc* mice, accelerates the emergence of Eµ-*Myc*-induced lymphomas (14). A similar effect was shown on an EBV dependent mouse B-cell lymphoma (10), and also in a BL λ-*Myc*-*Klrk1^-/-^* mouse model (15). Belting *et al*. completed this observation by demonstrating the role of NKG2D in the control of B-cell lymphoma growth and observed another escape mechanism developed by lymphomas cells through downregulating NKG2DL during the NK cell activation through downregulating NKG2DL (15).

All these different studies demonstrate the importance to escape the recognition of NKG2D receptor in B-cell lymphomagenesis and suggest its implication in the aggressive phenotype of these diseases.

## Conclusion

The pressure of the immune surveillance is certainly one of the major driving forces in the emergence of B-cell lymphomas. Together, results reported in the articles reviewed here, support the fact that mouse models are useful to analytically understand the immune escape in both aggressive and indolent B-cell lymphomas. Beyond PD-1/PD-L1 axis, roles of CTLA-4, of antigen presentation and of NK-cells is also highlighted. These models functionally explain and resume the role of the different actors of the immune surveillance in B-cell lymphomas. Engineered mouse models will contribute to better understand tumor microenvironment in the aim to identify novel mechanisms of B-cell immune escape, which may be the keystone of future therapies. Mouse models are also useful for the preclinical study of these therapies, evidencing the interest of combinations that include treatments able to restore the anti-tumor immune response. All the models presented here point out the major role of NF-κB activation, suggesting that this pathway should also be targeted together with immune restoration therapies. Before trying such strategies in Humans, this is testable in genetically engineered mouse models. Providing a strong support for the issue of immune escape in B-cell lymphomas, these models deserve to be widely used.

## Author Contributions

All authors contributed to the article and approved the submitted version. QL performed the literature review and prepared the figure and table. QL, HA, JF, and CVF wrote the manuscript.

## FUNDING

The group of JF is supported by grants from the Ligue Nationale Contre le Cancer (Equipe labellisée Ligue), the Comité Orientation Recherche Cancer (CORC), the France Lymphome Espoir association, the Nouvelle Aquitaine Region and the Haute-Vienne and Corrèze committees of the Ligue Nationale Contre le Cancer. CVF was supported by the France Lymphome Espoir association of patients. QL was supported by the Fondation pour la Recherche Médicale (FRM, ECO202006011493).

## Conflict of Interest

The authors declare that the research was conducted in the absence of any commercial or financial relationships that could be construed as a potential conflict of interest.
